# Early laparotomy and timely reconstruction for patients with abdominal electrical injury: Five Case Reports and Literature Review

**DOI:** 10.1097/MD.0000000000007437

**Published:** 2017-07-21

**Authors:** Pi-Hong Zhang, Zan Liu, Li-Cheng Ren, Ji-Zhang Zeng, Geng-Wen Huang, Mu-Zhang Xiao, Jie Zhou, Peng-Fei Liang, Ming-Hua Zhang, Xiao-Yuan Huang

**Affiliations:** a Department of Burns and Reconstructive Surgery; b Department of General Surgery; c Institute of Burn Research, Xiangya Hospital, Central South University, Changsha, Hunan Province, P.R. China.

**Keywords:** abdominal-wall defect, electrical injury, exploratory laparotomy, flap, reconstruction

## Abstract

**Introduction:**

High-tension electricity can cause devastating injuries that may result in abdominal wall loss, visceral damage, and sometimes major threat to life. The visceral organ may be exposed after debridement and require flap cover, but the tensile strength of abdominal wall may be lack even if flap transplanted.

**Methods:**

From April 2007 through May 2015, 5 patients with severe abdominal electrical injury were treated at our hospital. Exploratory laparotomy was performed based on their clinical manifestations and debridement findings of abdominal wall at early stage, and decision regarding technique for reconstruction of abdominal wall was based on an assessment of the location and extent of the defect. Medical records were reviewed for these data.

**Results:**

Clinical evaluation and debridement findings of the abdomen revealed 4 patients with suspicious visceral damage. Laparotomy was performed in 4 cases, and revealed obvious lesion in 3 cases, including segmental necrosis of small intestine, partial necrosis of diaphragm, left liver and gastric wall, and greater omentum. Five patients underwent abdominal wall reconstruction using island retrograde latissimus dorsi myocutaneous flap or free/island composite anterolateral thigh myocutaneous flap. All flaps survived, abdominal bulging occurred in 3 cases after follow-up of 12 to 36 months.

**Conclusions:**

The clinical manifestations and wound features of abdomen collectively suggest a possible requirement of laparotomy for severe abdominal electrical burns. Retrograde latissimus dorsi myocutaneous flap or composite anterolateral thigh myocutaneous flap is an effective option for reconstruction of abdominal wall loss, the long-term complication of abdominal bulging, however, remains a significant clinical challenge.

## 1 Introduction

Electrical injuries on abdominal wall are uncommon, but associated with high mortality, especially delayed gastrointestinal perforation and peritonitis complicated after the visceral organ injury.^[1,2]^ The prognosis of visceral organ injury is related to early diagnosis and proper management, despite early recognition of the visceral damage is quite difficult.^[3]^ Exploratory laparotomy can find ischemic or necrotic tissue, perforate hollow organ, and other damage, but sometimes no obvious lesion can be found after laparotomy,^[4]^ the indication of exploratory laparotomy is still deserved to be investigated.

The basic principle of electrical burns, especially visceral organ exposed after debridement, is to repair the defect with a vascularized flap.^[5–7]^ A series of free or pedicled anterolateral thigh (ALT) and/or tensor fasciae latae (TFL) flaps can be chosen for abdominal wall reconstruction depending on the size and location of the defect and the length of the vascular pedicle.^[8–17]^ The benefits of flap cover in the acute stage are early wound coverage, preservation of vital viscera and digestive function, reduced invasive infection, and hospital stay.^[18]^ But the tensile strength of abdominal wall may be lack even if flap transplanted, a flap with strong, vascularized fascia and/or innervated muscle can enhance the tensile strength of abdominal wall to avoid abdominal hernia formation .^[3,10,15,19–20]^ Therefore, the reconstruction of large defects on the abdominal wall after electrical injury can be challenging. The purpose of this article is to retrospectively analyze the characteristics of abdominal electrical injury with visceral damage and to evaluate the outcome of early flap repair of large defects on the abdominal wall after electrical burns.

## 2 Patients and methods

### 2.1 Data of patients and consent

Five patients suffering from a high-voltage electrical injury to extensive abdominal wall were treated at our academic hospital from April 2007 to May 2015. All of the patients were male with an average age of 46 years (range, 35–54 years). The induced voltage of these patients was 1000 to 100,000 V and they were evaluated on admission between 16 and 24 hours (mean 19 hours) after burn injury. None of the patients had any chronic illnesses, such as diabetes or cardiovascular insufficiency, nor did they have cardiogenic or hypovolemic shock symptoms or experience other major trauma, such as an intracranial injury or a bone fracture. The majority of patients’ limbs were injured or even damaged, and the calculated total burned body surface area of these patients ranged from 3% to 19% (mean 11.0%). The size of abdominal wall wound ranged from 360 to 700 cm^2^ (mean 494.4 cm^2^). After admitted, meticulous symptom inquiry and detailed physical examination, if needed, serial accessory examination such as abdominocentesis, KUB, ultrasonographic examination, and so on were performed. Besides, 4000 to 6000 mL fluid was administrated for fluid replacement to maintain a clear hourly urine output to 50 to 75 mL/h.

Consents to conduct and report of this study were obtained from the Ethics Committee of Xiangya Hospital, Central South University. Written informed consent for publication of this case report and any accompanying images was obtained from the patient.

### 2.2 Debridement and laparotomy

Debridement was performed within 1 to 3 days after injured. Eschar and obvious devitalized muscle or other tissues were removed, the appearance of viable abdominal muscle, posterior rectus sheath, or peritoneum were noted and left as much as possible during debridement. All patients were debrided for one time. According to the clinical manifestations mentioned above and debridement findings at early stage, laparotomy operations were performed for those under suspicion with internal organs injury. During laparotomy intestinal resection and anastomosis, or resection of necrotic tissues (eg, greater omentum and gastric, etc) and repair of visceral organ were performed, and the peritoneal cavity was closed with peritoneum or greater omentum.

### 2.3 Flap transplantation and follow-up

After debridement and laparotomy, one-stage reconstruction followed. The defect of abdominal wall was repaired with island retrograde latissimus dorsi (LD) musculocutaneous flap, free, or island composite ALT musculocutaneous flap. The selection of flap for abdominal wall reconstruction was determined by the location and size of the defect. During harvest of the former perfused by lumbar artery and perforator intercostal vessels, a major perforator from the 10th intercostal vessels was secured. When harvesting of the latter perfused by the lateral circumflex system of femoral vessels, vastus lateralis, or rectus femoris and the femoral nerve of these muscle were carried if necessary. The deep inferior epigastric vessels were used as the recipients for end-to-end anastomoses during free-flap transplantation. To strengthen the abdominal wall, the carried vastus lateralis or rectus femoris was sutured with remnant rim of abdominal muscles including the remaining rectus abdominis using numerous strong prolene sutures, the fusion of fascia lata was fixed to the external oblique aponeurosis under suitable tension, and the femoral nerve of vastus lateralis muscle was coapted with the intercostal nerve when free transplanted. The immediate and long-term outcomes were assessed regarding the complications and sustainable strength of the abdominal wall. The patient characteristics are shown in Table 1.

**Table 1:**
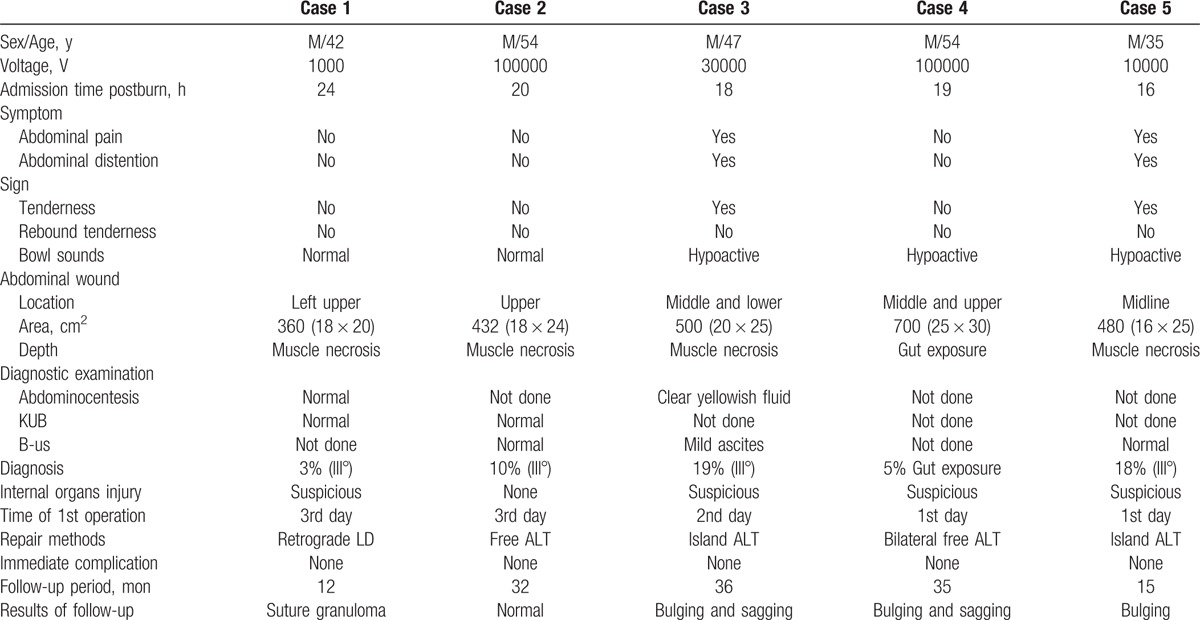
Characteristics of 5 patients with electrical injury on abdomen.

## 3 Case presentation

### 3.1 Case 1

**Figure 1:**
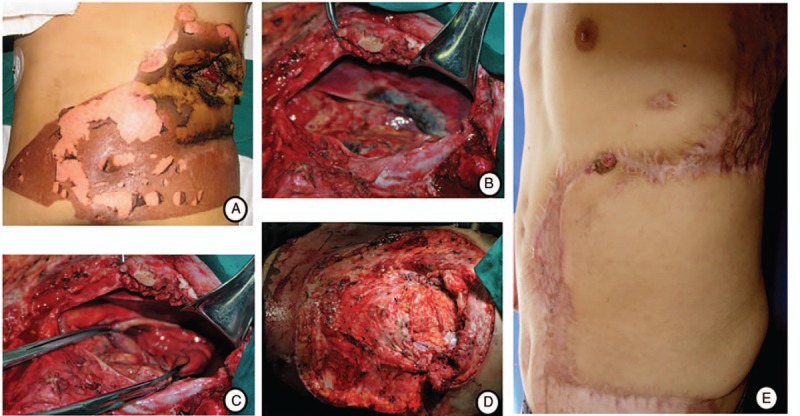
A, High-voltage electrical injury on the left upper quadrant of abdomen and lumber region; (B–C) Necrosis of the diaphragm and anterior gastric wall was showed after laparotomy; (D) Abdominal wound before flap transplantation. E, Twelve months after repair with retrograde LD musculocutaneous flap, a suture granuloma was seen on the surgical incision.

On physical examination, an abdominal and lumber burn wound of about 20 cm × 15 cm was detected, and we found a direct deep wound to the liver and stomach surface through the necrotic wound of the left 9th and 10th ribs, a 2 cm × 3 cm area of necrosis of the diaphragm, marginal necrosis of left liver and a necrotic pitting in the anterior wall of stomach with no obvious perforation during debridement. After resection and repair of injured diaphragm and gastric wall, we used retrograde LD musculocutaneous flap to cover the defect (Fig. 1).

### 3.2 Case 2

**Figure 2:**
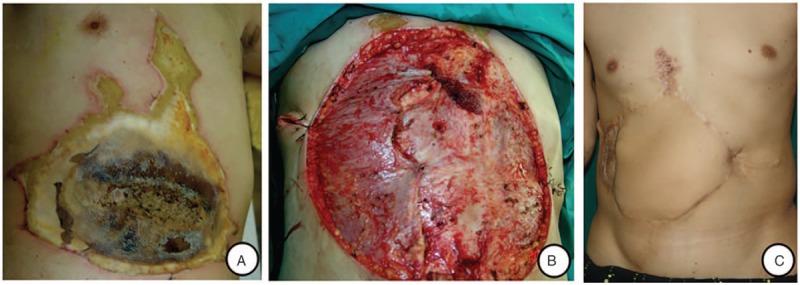
A, Full-thickness injury of the abdominal wall after injury; (B) abdominal defect after debridement; and (C) 32 months after the reconstruction of the abdominal wall defect with free composite ALT musculocutaneous flap and split-thickness skin graft.

During the abdominal debridement of case 2, bilateral rectus abdominis necrosis was revealed, but posterior rectus sheath and peritoneum however remained intact. To prevent secondary necrosis of the wound tissue, skin graft failure and hernia formation, free composite ALT flap with vastus lateralis was used to close the abdominal wall defect, the femoral nerve of vastus lateralis muscle was coapted with the intercostal nerve (Fig. 2).

### 3.3 Case 3

**Figure 3:**
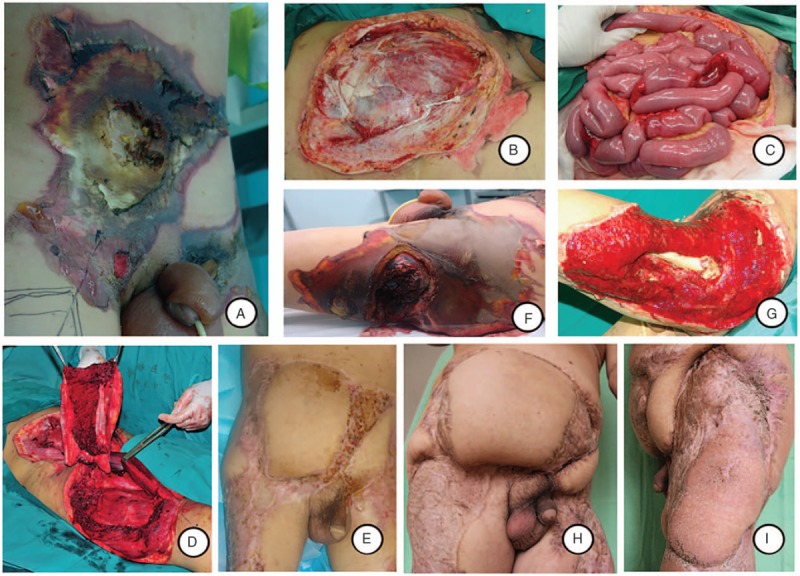
A, Abdominal lesions 18 hours after injury; (B) abdominal defect after debridement; (C) segmental intestinal devitalization during laparotomy; (D) harvest of island composite ALT musculocutaneous flap; (E) one and a half months after the flap has healed, the abdominal wall was stable; (F) wound on the left thigh and hip; (G) femur exposed after serial debridement and negative pressure wound therapy; (H-I) 36 months after the reconstruction of the abdominal wall and left thigh defect with island composite ALT musculocutaneous flap and free LD musculocutaneous flap.

In case 3, we found wide devitalized abdominal muscles with intact posterior sheaths of bilateral rectus abdominis and peritoneum after the emergency abdominal debridement. Laparotomy was still performed considering his positive abdominal signs and symptoms, and abdominal ascites confirmed by abdominal sonography and abdominocentesis before debridement. In spite of the peritoneal completeness, laparotomy revealed that 7 burn lesions in the range of 1.2 to 2.2 m below Treitz ligament were involved, 4 of which (1.2, 1.5, 2.1, and 2.2 m, respectively) presented with pitting and contracture of the intestine, and edema was noted in the remaining three sites. After the repair of the 3 edematous lesions, the necrotic intestines were resected and followed by enteroanastomosis. Right island composite ALT musculocutaneous flap was designed to close the abdominal defect. The abdominal wall was strengthened by suturing up the vastus lateralis with the rectus abdominis, and the fusion of fascia lata with the external oblique aponeurosis. The residual defects were covered by split-thickness skin grafts. On day 8 in hospital, the patient underwent amputation of the left forearm and the right arm, and debridement of the left thigh. On day 26 in hospital, free LD musculocutaneous flap was designed to cover the defect with bone exposure in the left thigh. Thirty-six months after repair, the reconstructed abdominal wall was bulging without herniation of intraperitoneal organs (Fig. 3).

### 3.4 Case 4

**Figure 4:**
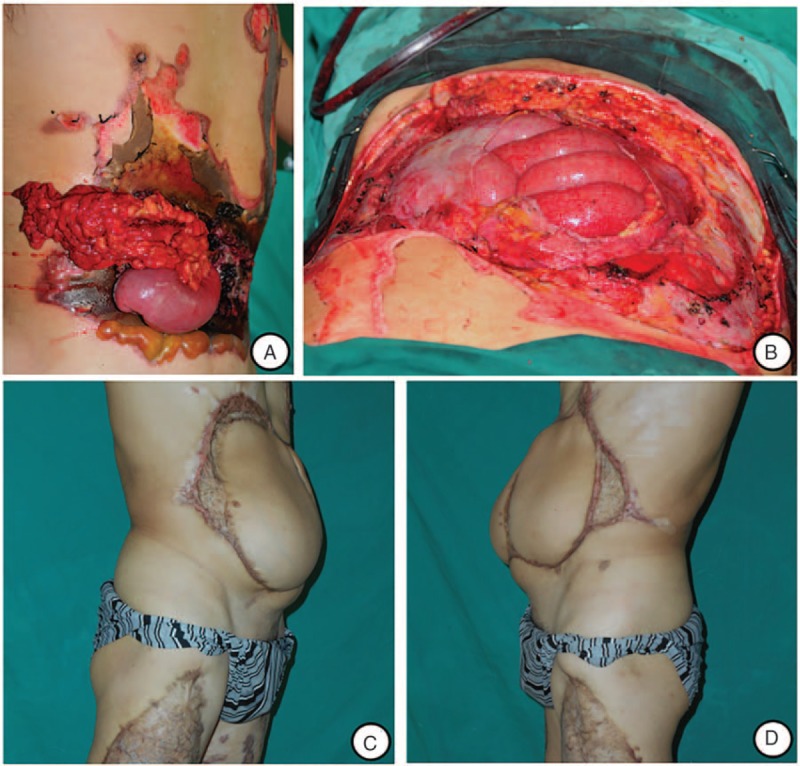
A, Abdominal wound and gut exposure after injury; (B) abdominal defect after debridement; (C–D) 35 months after the reconstruction of the abdominal wall defect with bilateral free composite ALT flap.

To case 4, an emergency exploratory laparotomy was done owing to gut exposure after abdominal debridement. Although posterior sheath and peritoneum were necrotized, almost normal except partial greater omentum necrosis was found. Bilateral free composite ALT flap with a cuff of vastus lateralis was used to repair the defect of abdominal wall after necrotic omentum was resected. 35 months after repair, the reconstructed abdominal wall was bulging without herniation of intraperitoneal organs (Fig. 4).

### 3.5 Case 5

**Figure 5:**
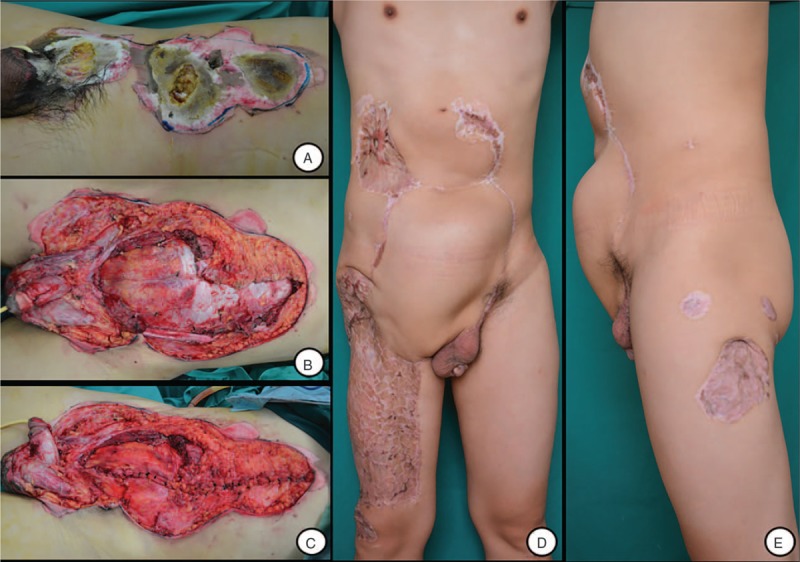
A, Abdominal wound after injury; (B) defect after debridement; (C) defect after laparotomy; (D–E) 15 months after reconstruction using island composite ALT with innervated rectus femoris muscle, distinct abdominal wall bulging in the left wound which repaired without innervated muscles.

We found wide devitalized abdominal muscles with intact posterior sheaths of bilateral rectus abdominis and peritoneum after the emergency abdominal debridement and only flatulence after laparotomy. A giant composite ALT flap with innervated rectus femoris was transplanted to cover the abdominal wall after manual exhaust and decompression. (Fig. 5)

## 4 Results

Exploratory laparotomy was performed in 4 cases, and revealed small pieces of necrosis on the diaphragm, left liver and the anterior wall of stomach in 1 case, segmental necrosis of small intestine in 1 case, partial greater omentum necrosis in 1 case, and no obvious lesion except large intestine distension in 1 case. All patients underwent abdominal wall reconstruction using retrograde LD musculocutaneous flap or composite ALT musculocutaneous flap, pedicled in 3 cases and free in 2 cases. Complete flap survival was achieved without partial necrosis or thrombosis. The contraction forces of quadriceps femoris muscle was mildly reduced in case 2, 3, and 5; clinical evaluation of the abdomen revealed 2 patients without anomalies, 3 cases of abdominal bulging and sagging without functional discomfort but needing to wear an abdominal lap belt in 2 cases, and 1 case of suture granuloma.

## 5 Discussion

High-voltage electrical injury of the abdomen usually causes devitalization of the whole layers of the abdominal wall, even results in injuries of the internal organs. As acute peritonitis may increase the chances of wound infection and sepsis, and thus death rate, furthermore, it may also bring disadvantages to the following surgical and nursing care; reduce the patients’ quality of life. Exploratory laparotomy can find some visceral organ lesion, and flap coverage is of benefit to controlling the wound infection effectively. These 2 procedures need to be carried out before the situation of abdominal lesion being exacerbated. Therefore, early diagnosis of abdominal organ injuries, early exploratory laparotomy, and timely coverage of the abdominal wall defects are the keys to enhance the patients’ prognosis.

The indications for laparotomy after electrical injury on the abdominal wall still remain ambiguous, which is related with larvaceous visceral damage and delayed necrosis after electrical burns. To improve these patients’ prognosis, decision regarding laparotomy was based on their clinical manifestation and debridement findings of abdominal wall by us. Fortunately, significant visceral lesion was found through laparotomy in 3 cases. No obvious internal organ injury was found in case 4 and 5 even if doubtful manifestations of acute peritonitis or gut exposure existed after injury, and these phenomena pointed out the extent of internal organ injury was not proportional to the depth of wound after electrical injury. The segmental and jumping injury are the characteristic of electrical burns. We surmised the multiple intestinal segments necrosis may have been because of the flow of current through peritoneum into these roundabout bowels close to the anterior abdominal wall. In brief, we believed that laparotomy was important precautions after achieving on the correct assessment of clinical manifestation and debridement findings, to avoid delayed gastrointestinal perforation and peritonitis with potential visceral injury after electrical injury on abdominal wall.

In the light of conventional methods, a temporary abdominal closure can be accomplished with omentum or a variety of synthetic coverings, followed by skin grafting directly onto visceral granulation tissue, and pedicle or free myocutaneous flap should be repaired secondarily.^[20–23]^ However, such multiple stage reconstructions are time- and cost-consuming, and the results are usually not satisfying.^[24–25]^ Therefore, one-stage reconstruction with vascularized fascia has been proposed to overcome such disadvantages.^[16,20,26]^ Five patients in our report underwent early flap transplantation with all flap survival, wound primary healing, no recent complications, and short hospitalization period.

Abdominal wall reconstruction includes the reestablishment of the skin and fascia, and muscles and their aponeuroses to prevent hernia formation, and the main methods include the use of prosthetic (mesh) or autologous material (tissue flaps).^[27–30]^ To large defect of abdominal wall resulting from electrical injury, the former should be used with caution owing to the poor wound condition and high risk of infection.^[29–33]^ The selection of flap is influenced by the size and location of the defect and the vascular pedicle length of the flap, the 5 cases presented above with extensive defects on the various site of abdominal wall, and island retrograde LD musculocutaneous flap, free and island composite ALT musculocutaneous flap were used respectively. By means of our clinical practice, retrograde LD musculocutaneous flap can successfully repair unilateral defects of abdomen and lumber region that reach the vicinity of the middle line, and island composite ALT musculocutaneous flap can be used to cover defects of the middle and lower abdomen that are extended 6 to 8 cm above the umbilicus. More than 3% total body surface area wound of case 4 is on the middle and upper abdomen, the pedicled ALT flap is difficult to reach and cover the wound, bilateral free composite ALT flap was employed for reconstruction of the large defect. The cautious problem of electrical injury is secondary necrosis, high-failure of free flap is mainly owing to recipient vascular erosion,^[6]^ we use the deep inferior epigastric vessels 4 to 5 cm over the wound edge as the recipient vascular, and 3 free flaps mentioned above were successfully transplanted without vascular crisis.

Long-term follow-up showed 3 cases with abdominal bulging; however, surgical revision was not necessary because no functional discomfort occurred. The similar outcome of abdominal bulging has been reported by Bodin,^[19]^ and the significant risk of long-term complication should be advised to patient. In addition, possible donor site morbidity should also be considered. As Kuo et al^[18]^ reported quadriceps femoris muscle contraction forces of donor thigh was reduced, on average, by 30% after harvest of an ALT flap combined with vascular fascia lata (without fascia lata muscle) and a cuff of vastus lateralis muscle in spite of the fact that no difficulties in daily ambulating occurred. In consideration of the long-term outcomes of cases 3, 4, and 5, the majority of abdominal bulging could be considered to be dependent on the lack of muscle and fascia structure with tensile strength, some of which is associated with the swollen wound and disability obesity after electrical burns. Therefore, we suggest that some adjacent muscle may be carried with the ALT flap used to repair the defect on the middle or lower part of abdominal wall.

Previous publications about the reconstruction of abdominal wall defect with an innervated free LD or ALT musculocutaneous flap, the thoracodorsal nerve or femoral nerve of the vastus lateralis muscle was coapted with the intercostal nerve,^[3,19,34]^ but the recipient nerve is very difficult to find and not suit for anastomose with the nerve of flap owing to length and orientation in some cases. We used an innervated free ALT musculocutaneous flap in case 2, and pedicled innervated vastus lateralis or rectus femoris muscle in case 3 and 5, respectively, the result of follow-up is passable, especially distinct bulging appeared only in the left wound that repaired without innervated muscles in case 5, but the morbidity of donor-site is more obvious of course, and more extensive investigations with comparative studies are needed. By this token, the perfect reconstruction of abdominal wall loss after electrical injury still remains a significant clinical challenge.
